# A Case of Attempted Suicide by *Cerbera odollam* Seed Ingestion

**DOI:** 10.1155/2020/7367191

**Published:** 2020-06-15

**Authors:** Michelle Bernshteyn, Steven H. Adams, Kunal Gada

**Affiliations:** SUNY Upstate Medical University, 750 E Adams St., Syracuse, NY 13210, USA

## Abstract

We report a case of attempted suicide by *Cerbera odollam* seed ingestion by a transgender patient who was successfully treated at our hospital. While the *C. odollam* plant has multiple practical and ornamental functions, its seeds have traditionally been utilized for suicidal and homicidal purposes in many parts of the world. Physicians should be aware of the presentation, diagnosis, and treatment of *C. odollam* ingestion given the current ease of availability of these seeds in the United States and the increased reports of suicide attempts.

## 1. Introduction

Indigenous to India and Southeast Asia, *Cerbera odollam*, also known as pong-pong, or “suicide tree,” yields highly cardiotoxic seeds. These seeds are a common cause of suicide or homicide in their countries of origin [1]. The seed's primary toxic ingredient is cerberin, which causes disruption of cardiac electrical activity and hyperkalemia by inhibiting the Na-K-ATPase exchanger in myocardial cells. Until recently, the plant was hardly known or easily available in the United States. However, now, seeds of the tree, which have ornamental appeal, are available for sale online by multiple tropical plant retailers.

## 2. Case Presentation

We present a 32-year-old transgender female with a past medical history most significant for depression with suicidal ideation who presented to our hospital due to an acute onset of nausea and lethargy with one episode of nonbloody emesis. Symptoms were not alleviated or exacerbated by any specific factors. The patient had purchased a *Cerbera odollam* plant on the internet a week prior. She ingested one seed of this plant, as this was suggested to be the lethal dose, with the intent of ending her life. The exact time between ingestion and presentation was unknown. On transportation to the hospital, the patient had a documented heart rate in the 30s with a junctional rhythm and therefore received a total of 10 vials of Digibind (digoxin immune fab). She denied any headache, visual disturbances, chest pain, palpitations, shortness of breath, abdominal tenderness, diarrhea, or constipation.

On presentation, the patient's vital signs were blood pressure of 105/74 mmHg, heart rate of 106 beats per minute, respiratory rate of 18 breaths per minute, body temperature at 98.6 degrees F, and oxygen level of 98% on room air. Physical examination was noted as within normal limits. Labs performed included urine toxicology which was positive for cannabis, digoxin level 0.2 ng/mL, salicylate level less than 1.7 mg/dL, acetaminophen level less than 2 mcg/mL, blood alcohol content of 0, lactic acid 1.2 mmol/L, white blood cell count 8,100/*μ*L, hemoglobin 13.9 g/dL, hematocrit 40.8%, platelets 165/*μ*L, sodium 138 mmol/L, potassium 4.3 mmol/L, chloride 103 mmol/L, bicarbonate 29 mmol/L, blood urea nitrogen 10 mg/dL, creatinine 0.84 mg/dL, glucose 107 mg/dL, total bilirubin 0.5 mg/dL, AST 37 U/L, ALT 61 U/L, and alkaline phosphatase 48 U/L.

**Figure 1:**
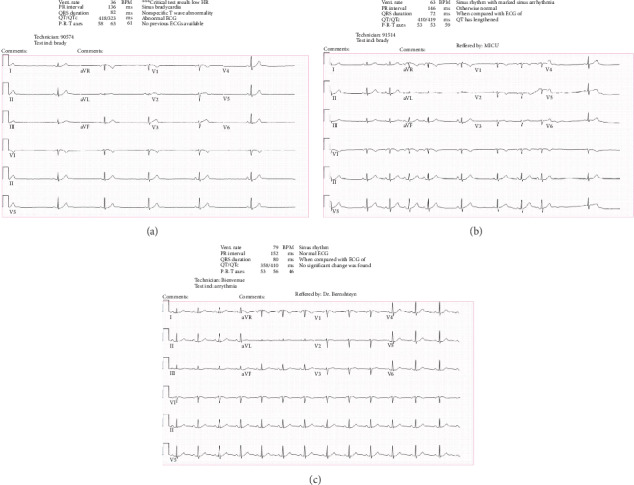
(a) Initial ECG taken at time 04:19 at our hospital demonstrating sinus bradycardia. (b) Repeat ECG at time 09:25 showing resolution of sinus bradycardia, but with marked sinus arrhythmia. (c) Repeat ECG at time 10:25 showing sinus rhythm and normal ECG.

Electrocardiogram (ECG) was performed shortly after arrival. This demonstrated a significant decrease in heart rate as compared to presenting vital signs. The heart rate had decreased from 106 beats per minute to 36 beats per minute (Figure 1(a)). Therefore, the patient was monitored on telemetry, and repeat ECGs were ordered. Her heart rate remained in the 30s to 40s, and the rhythm was sinus bradycardia. Upon consultation, the toxicology team recommended to monitor electrolytes and heart rate closely while continuing intravenous fluids. If there were any conduction blocks or further slowing of heart rate, 5 more vials of Digibind could be given. She had a hypoglycemic episode with blood glucose of 60 mg/dL. She was administered dextrose and responded appropriately.

The following day, the psychiatry team was consulted, and the patient was diagnosed with gender dysphoria and depressive disorder and noted to have narcissistic and borderline personality traits. The patient met the criteria for inpatient psychiatric hospitalization once medically cleared.

The toxicology team continued to follow the patient during her hospitalization. Being that her digoxin level was only 0.2 ng/mL and she had normalized potassium levels, her likelihood for toxicity was low. Given her lab work and improving heart rate on ECGs (Figures 1(b) and 1(c)), the team was reassured that the patient's clinical status was improving. The patient's home hormonal therapy was started, and she was admitted to the psychiatry unit.

## 3. Discussion

Members of the genus Cerbera, both *Cerbera odollam* (also referred to as *C. manghas* Linn., pong-pong, and “suicide tree”) and its close relative *Cerbera manghas* (sea mango), produce the cardiotoxin cerberin. Due to the toxicity of the seeds, the genus was named after Cerberus, the “hound of Hades,” a monstrous watchdog of the underworld that was said to have a poisonous bite in Greek mythology [2]. The *C. odollam* tree grows along seashores, rivers, and salt swamps of the tropics including southern India, Southeast Asia, Madagascar, and Australia [1]. *C. manghas* has a similar geographic distribution [3, 4]. *C. odollam* trees were introduced in the United States over a century ago in Hawaii [5]. *C. manghas* and *C. odollam* are similar in appearance (*C. manghas* flowers have a pink center, in contrast to the yellow center of *C. odollam* flowers) and have identical toxins and toxic effects. The clinical management for ingestion of both species is presumed to be the same [6].

The primary poisonous ingredient in the seed is cerberin, with a clinical presentation and mechanism of action like that of digoxin (of the plant *Digitalis purpura*), both cardiac glycosides. The reported presenting symptoms of *odollam* toxicity include headache, muscle weakness, dizziness, altered mental status, nausea, vomiting, abdominal pain, chest pain, and palpations. Bradycardia, hyperkalemia, and thrombocytopenia are often present. A wide variety of ECG findings are reported and consist of sinus bradycardia, sinus pauses, junctional rhythm, prominent U-waves, wandering pacemaker, atrial fibrillation, ventricular tachycardia, digoxin-like ST changes, nodal rhythm, and first-, second-, and third-degree heart block [1, 7–9]. According to a 2016 study of 50 *odollam* poisoning cases in India, the most frequent symptom is vomiting (54% of the studied patients); thrombocytopenia was observed in 50% of patients; the most common ECG abnormality was sinus bradycardia (32%), followed by sinus pause (28%) [10]. However, another study out of India of 102 patients reported varying degrees of heart block (58%) as the most common ECG finding [11]. Junctional rhythm seen in our patient is infrequently reported [9]. Death by *C. odollam* ingestion is described as painful [12].

The described mechanism of action for cerberin poisoning is by binding to and reversibly inhibiting the sodium-potassium adenosine triphosphatase (Na-K-ATPase) exchanger in cardiac cells, resulting in extracellular accumulation of potassium and intracellular accumulation of sodium. This disruption of the electrochemical gradient of sodium by increased intracellular sodium prevents the work of the passive sodium-calcium exchanger, an antiporter membrane protein that under normal physiological conditions removes calcium from cells utilizing the energy that is stored in the electrochemical gradient. Disruption of this exchanger causes intracellular calcium buildup, thereby lengthening the cardiac action potential, which leads to a decrease in the heart rate [6, 13]. In turn, increased cytoplasmic calcium facilitates increased calcium uptake to the sarcoplasmic reticulum (SR), which then allows for greater calcium release from the SR upon stimulation, resulting in increased inotropy [14]. The resultant accumulation of extracellular potassium is responsible for the described clinical findings of hyperkalemia. Hyperkalemia itself can be the cause of the findings of muscle weakness and arrhythmias.

Studies of the sea mango showed that a seed of the dried ripe fruit has a 285.9 *μ*g/g concentration of cerberin. A seed of the fresh unripe fruit had concentrations of 2.3 *μ*g/g [15]. Early animal studies found ingestion of a small amount of the seed to be fatal, with the lethal dose of cerberin at 1.8 mg/kg for a dog, 3.1 mg/kg for a cat, and 50 mg/kg for a rabbit [2]. Some studies report a minimum lethal dose for humans of half an *odollam* kernel, while others report that one full kernel is required for full lethal effect [11]. The body mass index of the consumer possibly determines the fatal amount [6]. In 102 patients studied by Renymol et al., consumption of more than two kernels was positively associated with mortality (OR of 5.12, CI of 1.54–17.04, and *P* = 0.004) [11]. Our patient with a BMI of 21.71 kg/m^2^ reported consuming one seed and survived.

**Figure 2:**
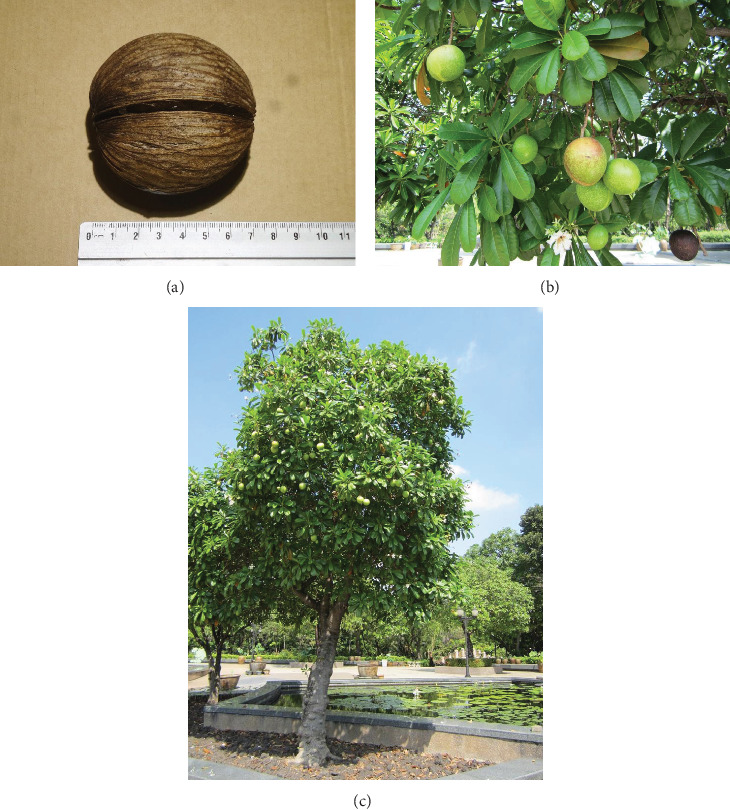
(a) *Cerbera odollam* seed. Image by Asean Plant Export Co. Ltd. Permission was obtained. (b) *Cerbera odollam* tree. Image by Asean Plant Export Co. Ltd. Permission was obtained. (c) *Cerbera odollam* tree. Image by Asean Plant Export Co. Ltd. Permission was obtained.

The plant's leaves, bark, and milky sap, said to be nontoxic, have been used as an emetic in traditional medicine [16]. The seed's high oil content, traditionally used for cosmetics, lamp oil, and insect repellent, is of recent being considered as a potential biodiesel source [2, 17–21]. Antinociceptive, antibacterial, and diuretic activities are found in extracts of *C. odollam* roots, leaves, and bark [22, 23]. The tree, which grows mango-like fruit, green fleshy leaves, and white flowers with a jasmine-like scent, is often planted as an ornamental tree (see Figures 2(a)–2(c)) [16]. Thus, procurement of seeds alone does not necessarily reveal fatal intentions.

While cerberin toxicity is rare in the United States, it is well known in the Asia-Pacific region. In the southwestern Indian state of Kerala, a single medical center alone reported 102 cases of *odollam* poisoning during the 2016 year [11]. Another study in Kerala showed that from 1989 to 1999, there were 537 reported deaths due to *odollam* poisoning [1]. Eating coconut crabs (*Birgut latro*) is reported as a frequent cause of secondary cerberin poisoning in islands of the South Pacific due to consumption of the Cerbera manghas fruit by the crustacean [24–27]. It is reported that in Asian communities, victims are more often women, often from traditional households [28]. Deaths, both homicides and suicides, are considered likely underreported, as upon autopsy, death by heart failure would be attributed to natural causes unless the pathologist had a reason to suspect poisoning [28]. The cerberin toxin cannot be detected by the digoxin level test. The method for identifying the *C. odollam* toxin cerberin is by ultraperformance liquid chromatography coupled with mass spectrometry [15, 28]. For our patient, a diagnosis of *C. odollam* poisoning, based upon the patient's history (admitted suicide attempt using “suicide tree” seeds) and hospital course (severe bradycardia, hyperkalemia), was considered sufficient. In 2014, Kassop et al. reported the first case in the United States of attempted suicide by *C. odollam* [29]. Since then, there appear to have been another eight or nine documented cases in the USA [7, 30]. Ours is the third reported case of *C. odollam* being used for suicide purposes in a transgender patient in the United States [7, 12].

Like digoxin toxicity, management of cerberin poisoning consists of supportive treatment of bradycardia and hyperkalemia as well as administration of digoxin immune fab. Studies have reported successful results with atropine and pacemaker therapy [10]. Digoxin immune fab, though, has had with mixed results, perhaps due to a lower affinity of the digoxin-specific antibody fragments for *C. odollam* toxins [29, 30]. Supportive treatment of hyperkalemia includes potassium-lowering agents with insulin-dextrose infusions. However, treatment of hyperkalemia has not been shown to reduce mortality [6].

Unfortunately, our female transgender patient with a history of major depressive disorder follows a disturbing pattern in the U.S. of suicide attempts by poisoning. 2017 data shows that 7.1% of U.S. adults have experienced one or more major depressive episodes in their lifetime, with a higher prevalence in females (8.7%) compared to males (5.3%) [31]. Depression is a strong risk factor for suicide [32]. This 32-year-old patient falls into a group aging 10–34 for which recent data reveals suicide to be the second leading cause of death. In 2017, 4.3% of U.S. adults reported thoughts of committing suicide. During that year, out of 47,173 suicide cases in the U.S., approximately 14% were accomplished by poisoning, which was the 3rd most used method after firearms and suffocation [33]. Our case further highlights the plight of members of the transgender community who are at increased suicide risk. According to the 2011 U.S. National Transgender Discrimination Survey, prevalence of self-reported lifetime suicide attempts amongst the 6,450 transgender and gender nonconforming study participants from 18 to 89 years of age was a staggering 41% compared to the 1.6% of the overall U.S. population [34].

## 4. Conclusion

This case should help raise awareness amongst physicians, chemists, coroners, and forensic toxicologists to the increasing incidence of cerberin poisoning attempts in the United States. From our experience, early involvement of poison control specialists is recommended. A case-by-case approach to cerberin toxicity is required as it may depend on factors such as quantity of seeds consumed, BMI, severity of hyperkalemia, and ECG changes, with varied response to treatment. The ease of online access to cerberin-containing seeds, which are available for as little as $5.00, is also a noteworthy concern [12]. It further brings attention to the outstanding predicament of suffering from suicidal ideation, depression, and other psychiatric conditions for many transgender patients.

## Data Availability

The data used to support the findings of this study are included within the article.
